# Expression of Human Kallikreins 4, 8, 10, 11 and 13 in Pleomorphic Adenomas and Mucoepidermoid Carcinomas

**Published:** 2016

**Authors:** Maryam Alsadat Hashemipour, Fatemeh Sadat Fatah, Mohammad Javad Ashraf, Mehrnaz Tahmasebi

**Affiliations:** 1 *Kerman Dental and Oral Diseases Research Center, School of Dentistry, Kerman University of Medical Sciences, Kerman, Iran*.; 2 *School of Dentistry, Shiraz University of Medical Sciences, Shiraz, Iran.*

**Keywords:** Human kallikreins, Benign, Malignant, Salivary gland tumors

## Abstract

**Background:**

In cancers of prostate, breast, oropharynx, lung, hypopharynx and skin, human tissue kallikreins has been demonstrated as a main role in these problems. There are many research works in which some human tissue kallikreins are expressed in salivary glands. In the present study, the main goal was to determine expression of human tissue kallikreins 4, 8, 10, 11 and 13 in pleomorphic adenomas and mucoepidermoid carcinomas.

**Methods:**

Sixty-six specimens (45 cases of pleomorphic adenomas and 21 cases mucoepidermoid carcinomas) were selected for final analysis by immunohistochemistry. For doing association test, clinical parameters obtained from the patients’ medical charts, which included age, gender were used and the nonparametric tests employed for statistical analyses.

**Results:**

The expression of human kallikreins 4, 8, 11 and 13 was more prominent in benign and malignant tumors compared to that in normal tissues and the difference was significant. In addition, the expression of human kallikreins 4, 8, 10 and 11 in malignant tumors was more than that in benign tumors, with statistically significant differences.

**Conclusion:**

The differences in the levels of human kallikreins 4, 8, 11 and 13 suggest that kallikreins may benefit in determining tumor behavior of salivary gland tumors.

## Introduction

Salivary gland tumors are considered important entities in oral medicine, pathology and maxillofacial surgery fields (1). They account for 2%–10% of all neoplasms of the head and neck region. Salivary gland tumors are probably the most histologically heterogeneous group of tumors with the greatest diversity of morphological and cellular features, making their classification very challenging (1,2). Taking into account a specific tumor into a single category is neither always straightforward nor reliable (1,3). 

These tumors can be divided in two benign and malignant groups. The majority of salivary gland tumors are benign and malignant salivary gland tumors are less frequent (1). The most common point of origin for salivary gland tumors is the parotid gland in up to 80% of all cases (2). For salivary gland tumors, different classifications were devised, such that these categories are continuously updated as new lesion arise (2). 

Salivary gland tumors are almost the main histologically heterogeneous group of tumors with probably the greatest morphological diversity and cellular features and considering a specific tumor into a single category is neither always reliable nor straightforward (4). Therefore, it is difficult for a skillful clinician to differentiate them. The diagnosis process can be a challenge because; one tumor is able to exhibit different morphological characteristics. Any mistakes in the diagnosis is equal to poor prognosis of the tumor, because these types of tumors exhibit diverse clinical behaviors in relation to recurrence, metastasis and treatment (1, 2). 

Currently new diagnostic methods like detecting cellular markers are used for differentiation, recurrence pattern determination, prognosis and treatment planning of these tumors. Some of these markers are human tissue kallikreins (hK) 6, 8 and p53 (5-7). Tissue kallikerins of human, which are a subfamily of serine proteases localized in tandem on human chromosome 19q13, are encoded by 15 gens (8-10). The first member of this protease subclass was identified in 1930s as an abundant protein in the pancreas. Kallikreins are overexpressed in ovarian, breast, and prostate carcinomas (8-13). 

Some human kallireins are expressed in salivary glands. Thereby, it is possible that some members of this family will be valuable markers for differentia diagnosis and patients monitoring with salivary gland cercinomas (6, 7, 12, 14). Also, kallikreins 4, 10 , 11 and 13 has been a prognostic factor in numerous human cancers, but its value as a prognostic factor in this histologically unhomogenous group of carcinomas has so far not been fully elucidated.

The purpose of present research work was to investigate the expression of kallikreins 4, 8, 10, 11 and 13 in pleomorphic adenomas and mucoepidermoid carcinomas.

## Materials and Methods

Sixty- six specimens (45 cases of pleomorphic adenomas and 21 cases mucoepidermoid carcinomas) were chosen for final analysis by applying immunohistochemistry method. Clinical parameters including age and gender, obtained from the patient's medical records were used for association tests. 

By applying a standard immunoperoxidase method (15), the samples of archival formalin-fixed, paraffin-embedded tumor tissues were cut in 5-micron sections and stained. The samples were gathered from different centers including the Department of Pathology, Dental School of Kerman, Kerman Shafa Hospital and Shiraz Namazi Hospital. A total of 45 pleomorphic adenomas (PA), 21 mucoepidermoid carcinomas (MEC) and 50 normal gland controls were used. Appropriate positive and negative (tissue slides with primary antibodies omitted) controls were used (Human KLK-8/Kallikrein-8 Human Cells Transfected Lysate (positive control), Human KLK-4/ Kallikrein-4 Human Cells Transfected Lysate (positive control), Kallikrein 13 transfected 293T cell lysat, Lysate from 293T cells transfected with Kallikrein 10 and KLK11 / Kallikrein-11 Human Cells Transfected Lysate) (Sino Biological Inc, China and Asia Chemi Tab Co, Tehran, Iran).

All of selected samples were examined under a microscope by two oral and maxillofacial pathologists who they did not have any inform from the pathologic diagnosis. Based on the published criteria in the WHO Classification of Tumors (16) and the Atlas of Tumor Pathology (17), the tumors were diagnosed. 

The human kallikrein (hK) 4, 8, 10, 11 and 13 rabbit polycolonal antibodies - raised against full-length hK 4, 8, 10, 11 and 13 using recombinant technology in a mammalian stable cell line system - were used at a dilution of 1:2000 (18). The recombinant hK 4, 8, 10, 11 and 13 were produced and purified by HPLC as described previously (7, 12, 19). This antibody is highly specific and has no detectable cross-reactivity with other kallikreins on western blots (20). 

The staining method was applied by deparaffinization in xylene for 13 min with two changes of xylene at room temperature, and consequently by transfer through graded alcohols and rehydration. For blocking the endogenous peroxidase, 3% H_2_O_2_ in methanol for 5 min was used. By a shaker, sections were rinsed in PBS for 10 min. For ten min, the slides were immersed in boiling citrate buffer (pH= 6.0) at high power and 10 min at 50% power in a microwave oven for getting the antigen retrieval. Then the samples were rinsed in water and PBS for 5 min, blocked in 10% horse serum in a humidified chamber for 30 min at room temperature and incubated with the hK 4, 8, 10, 11 and 13 primary antibodies for 60 min at room temperature. The samples were washed two times in PBS and then the biotinylated goat anti-rabbit secondary antibodies (1:200 dilution prepared in 10% horse serum) were applied at room temperature for 30 minutes at room temperature. After two rinses with PBS, the freshly prepared ABC reagent was applied for 30 min at room temperature. The enzymatic reactions were developed in a freshly prepared solution of 3, 39-diaminobenzidine tetrahydrochloride for 5 min. Next, the sections were rinsed with water, and for three min counterstained with hematoxylin, dehydrated, cleared with xylene, and finally mounted. A proportion and intensity scores were used by a well-documented system for obtaining hK 4, 8, 10, 11 and 13 immunostaining (21,22). 

The estimated fraction of positively staining normal gland or tumor cells was presented by the proportion score, where 0=none; 1<1/100; 2=1/100–1/10; 3=1/10–1/3; 4=1/3–2/3; and 5>2/3. 

The score is represented for staining intensity by the estimated average staining intensity of positively staining normal gland or tumor cells. For this purpose, 0=none; 1=weak; 2=intermediate; 3=strong. The total value of positive staining were then defined as the sum of intensity and proportion scores (ranges =0 for negative staining and =2–8 for positive staining). For all of the ductal or duct-like, non-ductal, and non-duct-like elements, scores were added and for each entity, an average was calculated (7). Ductal and acinar cells were scored in normal salivary gland tissue, separately. The cells lining were scored both duct-like structures and non-duct-like cells for the extent of positive and intensity of staining. To reach this goal, duct-like cells were considered as the ones that lined the lumens of duct-like structures within tumor tissue, such that non-duct-like cells did not exist in the tumor tissue that was not obviously lining the ducts. The squamous, mucous and intermediate cells were scored for MEC, while non-epithelial cells were not scored. Two separate examiners were used for staining to achieve consistency by comparison and correlation of assessments for having less inter-examiner variability.

For analyzing data, the nonparametricis tests were employed and the criteria P<0.05 is considered to gain statistical significance. To reach this goal, SPSS 18 software (Chicago, IL, USA) was used for numerical computations. 

## Results

Sixty-six specimens (45 cases of pleomorphic adenomas and 21 cases mucoepidermoid carcinomas) were investigated, and 40 cases (60.6%) were male. The mean age was 45.13±15.45 yr (males, 48.34±16.23 and females, 41.23±17.21) with an age range of 16-79 yr. Most cases were in the fourth, fifth and sixth decades of life with a peak incidence in the fifth decade. 53 tumors were located in major salivary glands (38 in the parotid, 12 in the submandibular, and 3 in the sublingual salivary glands) and 13 tumors in minor salivary glands. The mean ages of patients with the minor and major neoplasms were 41.24±13.51 and 53.55±12.45, respectively. 

***Kallikrein 4 expression profile***

Normal salivary gland tissues exhibited immunoreactivity to kallikrein 4, which was localized to the cellular cytoplasm of both ductal and non-ductal cells. Ductal cells showed higher staining intensity for kallikrein 4 compared to non-ductal cells. In PA, immunoreactivity to kallikrein 4 was observed in both the ductal and non-ductal cells, where ductal cells stained more intensely than non-ductal cells. PA sections exhibited consistently higher levels of kallikrein 4 than normal control tissues, which was significant (*P*=0.004) (Table1). 

**Table 1:** Staining in all cells including ductal and non-ductal ones and total scores for immunostaining of pleomorphic adenoma, for kallikrein 4 in human (mean average staining ± SD

**Asymp. ** * **sig. (2 tailed)**	**Average of total scores**	**Acinar and Non- ductal cells**			**Ductal/duct-like cells**				
		**Total**	**I** c	**P** b	**Total**	**I** c	**P** b	**No** a	
	**6.6±0.8**	**6.6±0.7**	**2±0.5**	**4.6±0.2**	**6.9±1**	**2.3±0.7**	**4.6±0.3**	**50**	**Normal**
**0.004** *	**7.2±0.75**	**6.9±0.7**	**2.1±0.3**	**4.8±0.4**	**7.5±0.8**	**2.5±0.4**	**5±0.4**	**45**	**PA**

PA: pleomorphic adenoma,

a : Number of samples,

b : Proportion score,

c : Intensity score,

* : Means with control group differ significantly (*P*<0.05)

***Kallikrein 8 expression profile***

Normal salivary gland tissues showed immunoreactivity to kallikrein 8 in both ductal and non-ductal

 cells (non-ductal cells>ductal cells). PA sections showed consistently higher levels of kallikrein 8 compared to normal tissue, which was significant (*P*=0.001) (Table 2).

**Table 2:** Staining in ductal cells and non-ductal cells and total scores for immunostaining of pleomorphic adenomafor human kallikrein 8 (Average staining ± SD)

**Asymp. ** * **sig. (2 tailed)**	**Average of total scores **	**Acinar and Non- ductal cells**			**Ductal/duct-like cells**				
		**Total**	**I** c	**P** b	**Total**	**I** c	**P** b	**No** a	
	**5±0.4**	**5.2±0.7**	**1.8±0.4**	**3.4±0.3**	**4.8±1**	**1.4±0.6**	**3.4±0.4**	**50**	**Normal**
**0.001** *	**7.2±0.6**	**7.0±0.6**	**2.3±0.4**	**4.7±0.2**	**7.4±0.6**	**2.6±0.4**	**4.8±0.2**	**45**	**PA**

PA: pleomorphic adenoma,

a : Number of samples,

b : Proportion score,

c : Intensity score,

* : Means with control group differ significantly (*P*<0.05)

***Kallikrein 10 expression profile***

The ductal cells structures showed higher staining intensity for kallikrein10 than non-ductal cells in the normal tissue. In PA for kallikrein10 expression, ductal cells were stained more intensely than non-ductal cells and the difference was significant (Table 3).

**Table 3:** Staining in ductal cells and non-ductal cells and total scores for immunostaining of pleomorphic adenomafor human kallikrein 10 (Average staining ± SD

**Asymp. ** * **sig. (2 tailed)**	**Average of total scores **	**Acinar and Non- ductal cells**			**Ductal/duct-like cells**				
		**Total**	**I** c	**P** b	**Total**	**I** c	**P** b	**No** a	
	**5.8±0.8**	**5.4±0.8**	**1.9±0.2**	**3.5±0.6**	**6.2±0.8**	**2±0.2**	**4.2±0.6**	**50**	**Normal**
**0.002** *	**7.1±1**	**7.0±0.7**	**2.5±0.2**	**4.5±0.5**	**7.2±1.4**	**2.7±0.7**	**4.5±0.7**	**45**	**PA**

PA: pleomorphic adenoma,

a : Number of samples,

b : Proportion score,

c : Intensity score,

*: Means with control group differ significantly (*P*<0.05)

***Kallikrein 11 expression profile***

Normal salivary gland tissues exhibited immunoreactivity to kallikrein11, which was localized to the cellular cytoplasm of both ductal and non-ductal cells. Ductal cells and cells lining duct-like structures showed higher staining intensity for kallikrein11 compared to non-ductal cells. In PA, immunoreactivity to kallikrein11 was observed in both the ductal and non-ductal cells, where ductal cells stained more intensely than non-ductal cells (Table 4). 

**Table 4:** Staining in ductal cells and non-ductal cells and total scores for immunostaining of pleomorphic adenomafor human kallikrein 11 (Average staining ± SD)

**Asymp. ** * **sig. (2 tailed)**	**Average of total scores **	**Acinar and Non- ductal cells**			**Ductal/duct-like cells**				
		**Total**	**I** c	**Pb**	**Total**	**I** c	**P** b	**No** a	
	**6.2±0.9**	**6.1±0.8**	**2.1±0.3**	**4.5±0.5**	**6.4±1**	**2.3±0.5**	**4.1±0.7**	**50**	**Normal **
**0.005** *	**7±0.7**	**7.0±0.8**	**2.4±0.5**	**4.6±0.3**	**7.1±0.6**	**2.5±0.3**	**4.6±0.3**	**45**	**PA**

PA: pleomorphic adenoma,

a : Number of samples,

b : Proportion score,

c : Intensity score,

*: Means with control group differ significantly (*P*<0.05)

***Kallikrein 13 expression profile***

This study revealed that normal salivary gland tissues showed immunoreactivity to kallikrein 13, which was localized to the cellular cytoplasm of both ductal and non-ductal cells. The ductal cells showed an overall higher staining intensity for kallikrein 13 compared to non-ductal cells. PA sections showed consistently higher levels of kallikrein 13 than normal control tissues (6.6 vs. 7.2, *P*=0.004) (Table 5).

**Table 5:** Staining in ductal cells and non-ductal cells and total scores for immunostaining of pleomorphic adenomafor human kallikrein 13 (Average staining ± SD

**Asymp. ** * **sig. (2 tailed)**	**Average of total scores**	**Acinar and Non- ductal cells**			**Ductal/duct-like cells**				
		**Total**	**I** c	**P** b	**Total**	**I** c	**P** b	**No** a	
**0.09**	**6.6±0.8**	**6.6±0.7**	**2±0.5**	**4.6±0.2**	**6.9±1**	**2.3±0.7**	**4.6±0.3**	**50**	**Normal**
**0.004** *	**7.2±0.75**	**6.9±0.7**	**2.1±0.3**	**4.8±0.4**	**7.5±0.8**	**2.5±0.4**	**5±0.4**	**45**	**PA**

PA: pleomorphic adenoma,

a : Number of samples,

b : Proportion score,

c : Intensity score,

* : Means with control group differ significantly (*P*<0.05)

***Expression profiles of kallikreins 4, 8, 10,11 and 13 in mucoepidermoid carcinoma.***

In MEC, immunoreactivity to kallikrein 13 was observed in squamous, intermediate, and mucous cells. Intermediate cells had higher staining intensity for kallikrein 13 than mucous cells and squamous cells. Squamous cells and intermediate cells stained significantly higher than the normal gland. MEC sections showed consistently higher levels of kallikrein 13 than normal control tissues, which was significant (6.8 vs 5.9, *P*=0.003). Squamous cells had higher staining intensity for kallikrein 4 compared to mucous cells and intermediate cells. Squamous cells and intermediate cells stained significantly higher than the normal gland. Average staining of MEC sections showed consistently higher levels of kallikrein 4 than normal control tissues, which was significant (6.6 vs 5.9, *P*=0.003). 

Intermediate cells had higher staining intensity for kallikrein 8 compared to mucous cells and squamous cells. Squamous cells and intermediate cells stained significantly higher than the normal gland. Average staining of MEC sections showed consistently higher levels of kallikrein 8 than normal control tissues, which was significant (*P*=0.002). Mucous cells had higher staining intensity of kallikrein 10 compared to squamous cells and intermediate cells. Squamous, intermediate and mucous cells stained significantly higher than the normal gland. Average staining of MEC sections exhibited consistently higher levels of kallikrein 10 than normal control tissues (*P*=0.001) (Table 6). 

**Table 6:** KLK13, 4, 8, 10, 11staining in Mucoepidermoid carcinoma (Average staining ± SD

	**Squamous cells** a			**Mucous cells** a			**Intermediate cells** a				Asymp. * **sig. (2 tailed)**
	**P** b	**I** c	**Total**	**P** b	**I** c	**Total**	**P** b	**I** c	**Total**	**Average staining (SD)**	
**Normal**	**3.5±0.6**	**2.3±0.6**	**5.8±1.2**	**4.6±0.5**	**2.3±0.5**	**6.7±1**	**4.5±0.4**	**2.3±0.4**	**5.1±0.8**	**5.9±1**	
**KLK 13**	**4.3±0.2**	**2.4±0.7**	**6.7±0.9**	**4.3±0.4**	**2.5±0.5**	**6.8±0.9**	**4.6±0.5**	**2.4±0.4**	**7±0.9**	**6.8±0.9**	**0.003** *
**KLK 4**	**4.6±0.3**	**2.7±0.7**	**7.3±1**	**4.2±0.6**	**2.8±0.1**	**7±0.7**	**3.8±0.1**	**1.6±0.7**	**5.4±0.8**	**6.6±0.8**	**0.003** *
**KLK 8**	**4.3±0.8**	**2.4±0.5**	**6.7±1.3**	**4.3±0.1**	**2.5±0.5**	**6.8±0.6**	**5±0.5**	**2.9±0.1**	**7.9±0.6**	**7.1±0.8**	**0.002** *
**KLK 10**	**5±0.7**	**2.8±0.6**	**7.3±1.3**	**4.7±0.2**	**2.7±0.1**	**7.4±0.3**	**4.5±0.5**	**2.4±0.4**	**6.9±0.9**	**7.2±0.8**	**0.001** *
**KLK 11**	**5±0.4**	**2.7±0.4**	**7.7±0.8**	**5±0.1**	**2.6±0.4**	**7.6±0.5**	**4.3±0.2**	**2.3±0.5**	**6.6±0.7**	**7.3±0.6**	**0.001** *

a : Average±SD,

b : Proportion score,

c : Intensity score,

* : Means with control group differ significantly (*P*<0.05)

***Finally***

This study showed that expression of kallikreins 4, 8, 11 and 13 in peomorphic adenoma was more than that in normal tissues. In addition, expression of kallikreins 4, 8, 10, 11 and 13 in mucoepidermoid carcinoma was more than that in normal control. The expression of kallikreins 4, 8, 10 and 11 in mucoepidermoid carcinoma was more than that in peomorphic adenoma and this difference was significant (*P*=0.001, *P*=0.001, *P*=0.005, *P*=0.02).

## Discussion

Human tissue kallikreins, are secrete serine proteases with Phsyiological roles and diverse expression patterns. It should be noted that the primarily known for their clinical applicability as cancer biomarker implicates kallikreins in cell growth regulation, angiogensis, invasion and metastasis.

They individually promote neoplastic progression and/or in cascades with other proteases and kallikreins. Besides, they possibly represent attractive targets for therapeutic intervention (23).

The family has 15 genes encoding secretion of serine proteases, such that from the encoding proteins, prostate-specific antigen (PSA or hK3) is an important and obvious marker for prostate cancer (24). 

There are great kinds of kallikreins, such as kallikreins 6, 7, 8, 10, 11, 13 and 14, those are related with several types of malignancy (8-13,23,26,27). In this subject, one can recall the kallikrein 8 gene which is upregulated in ovarian cancer (8, 9). 

The results extracted from the present study showed that most of the salivary gland tumors have downregulated levels of expression of kallikrein 13. Kallikrein 13 staining was in PA and MEC, although the ductal cells showed an overall higher staining intensity for kallikrein 13 compared to non-ductal cells. Experesion kallikrein 13 in salivary gland tumors showed in several studies (31, 32). 

The presence of kallikrein 13 in salivary gland tissue was described previously (31). The hK13 was able to cleave the major components of the extracellular matrix and may play a role in tissue remodeling and/or tumor invasion and metastasis (31).

In most salivary gland tumors, high level of kallikrein 13 expression was exhibited (32). In addition, staining in PA was less than that seen in normal salivary gland tissues. MEC and ACI did not stain more than normal salivary gland tissue while ACC and AC did. In most tumors, ductal cells and cells lining duct-like structures showed a higher intensity of staining in comparison to non-ductal types. Tumors with only non-ductal cells exhibited cytoplasmic staining, too. 

The human kallikrein 13 protein is encoded by the kallikrein 13 gene, and its physiological role is unknown. Kallikrein 13 is expressed in many normal tissues. It is upregulated in female genital organs but is dramatically downregulated in metastatic breast carcinoma cells and prostate, in comparison to primary carcinoma cells or normal cells (23, 19). In addition, overexpression of kallikrein 13 transcripts and/or proteins in ovarian carcinoma tissues, cell lines and/or serum and tumor ascites fluid has been demonstrated in several studies (23, 28-30). To date, there have been a few reports linking HK13 to cancer (28).

Similar to the findings by other studies (7, 23,9 ), PA sections revealed consistently higher levels of kallikrein 8 than normal tissues. In this study, PA sections showed consistently higher levels of kallikrein 8 than normal tissues that is similar to some study (7,23,9). 

Most salivary gland tumors have high levels of kallikreins 8 expression in comparison to total scores of normal salivary gland tissue and all malignant salivary gland tumors, because of MEC and adenocarcinoma (7). In addition, KLK8 was present in relatively high levels in ductal cells, as well as in non-ductal cells, of normal salivary gland tissues and benign and malignant salivary gland tumors that is` similar to our study. KLK8 was significantly higher in ductal cells than in non-ductal cells, and in general, malignant tumors expressed higher levels of KLK8 compared to normal salivary gland tissue and benign tumors.

Kallikrein 8 is found in high levels in normal tissue extracts of esophagus and skin and in fetal tissue extracts of ureters and tonsils, while it is present in lower levels in lymph nodes, testes, tonsils, kidneys, breasts, salivary glands, and fallopian tubes. 

Kallikrein 8 is also found in high concentrations in breast milk as well as in other biological fluids such as follicular and amniotic fluid, serum, seminal and cerebrospinal fluid (7, 23). 

Kallikrein 8 presents in high levels in ascites fluid and serum of ovarian cancer patients and propose it as a novel biomarker for ovarian cancer (9). Kallikrein 8 is downregulated in breast cancer tissues and cell lines (11, 23). Kallikrein 8 is over-expressed in cervical cancer cell lines with a high rate (33). Kallikrein 8 appears to suppress tumor cell invasiveness in non-small cell lung cancer by degrading fibronectin. It also retards cancer cell motility via inhibiting actin polymerization. In a mouse model, kallikrein 8 suppressed growth of tumor and invasion in vivo. In normal physiology and cancer, tissue kallikreins have now been implicated in proteolytic cascade pathways. These cascades are operative and regulatory in semen liquefaction, skin desquamation, psoriasis, skin cancer and prostate cancer. It leads to this fact that a proteolytic cascade involving kallikrein 8 and other kallikreins may be involved in salivary gland cancers (7). Kallikrein 10 is a human protein, which is one of the fifteen-kallikrein subfamily members located in a cluster on chromosome 19. Its encoded protein may have a great effect on the suppression of tumorigenesis in prostate and breast cancers (34).

Kallikrein 10 is found in high levels in cancers of breast, colon, ovaries, pancreas, prostate, head and neck, testes and in leukemia (23). 

Darling et al. (35) found that KLK10 levels in the Pleomorphic adenomas are significantly lower than those are in control tissues. Neither of the mucoepidermoid carcinoma showed a significant alteration in the immunoreactive scores of KLK10 in comparison with the normal salivary gland tissues. In our` study, the ductal cell structures showed higher staining intensity for KLK10 than non-ductal cells in normal tissue and in pleomorphic adenomas and mucoepidermoid carcinoma, ductal cells were stained more intensely than non-ductal cells and the difference was significant. 

Immunoreactivity to kallikrein 11 exists in both the ductal and non-ductal cells. Besides the present study shows that in comparison to non-ductal cells, ductal cells have higher staining intensity for kallikrein 4 . Immunoreactivity to kallikrein 11 exists in both the ductal and non-ductal cells. Besides, ductal cells have higher staining intensity for kallikrein 4 compared to non-ductal cells.

Expression of kallikreins 4 and 11 in salivary gland tumors has not been evaluated. Thereby the aim of this study was to determine whether hK4 and 10 is expressed in salivary gland tissues and salivary gland tumors, in order to compare normal tissues with tumor. 

Kallikreins are overexpressed in ovarian, breast and prostatic carcinomas and that some may be important new biomarkers for diagnosis and monitoring of many cancer types (9, 10).The overexpression of kallikreins in malignant tumors has been linked with both favorable and poor patient prognosis (6).

About hK4 and 11 expression in salivary gland tumors, this is the first report. There are many other kallikreins, such as hK5, hK6, hK7, hK8, hK10, hK11 and hK14, which are in association with various forms of malignancy (1, 2, 5, 6, 9). 

Most human kallikreins are expressed in the salivary glands (1, 2, 11, 12). Consequently, some members of this family may be valuable markers for differential diagnosis, sub- typing and monitoring of patients who have salivary gland carcinomas. 

In this paper for the first time, high expression of hK4 and 11 in many salivary gland tumors is reported. This isn't unexpected, as hK4 and 11 is a secreted protein expressed in glandular tissues (12), but further studies are required to determine whether the hK4 and 11 gene is up-regulated in salivary gland tumors and to determine the correlation with clinical outcome. As a necessary action, the measurement of serum levels of hK4 and 11 is required for achieving whether it can be used as a serum marker to monitor salivary gland tumors.

Kallikrein 4 is a recently identified member of the kallikrein family, regulated by androgens and its expression is highly specific for prostate. The gene product of hK4 is the first member of the kallikrein family, which is intracellularly localized; hK4 expression is regulated by estrogen and progesterone in prostate cancer cells (36). Furthermore, kallikrein 4 is reported in high levels in ovarian cancer (23). 

Human kallikrein 11 is expressed in a variety of normal tissues and fluids, including prostate, stomach, trachea, skin, and colon tissues and milk, serum, and seminal fluids (37, 38). 

Human kallikrein 11 has a relation to the prognosis of some human cancers, but in the steps of cancer progression, its physiological functions are still unknown.

Recent studies revealed kallikrein 11 expression in prostate and breast cancer cell lines and in ovarian, prostate, breast, lung, pancreatic, colon, and neuroendocrine cancer tissue (39-41).

Squamous cells from MEC frequently express hK4. hK4 have a functional role in the progression of prostate cancer through their promotion of tumour cell migration and increase in motility (42). Besides, hK4 may have a relation to the observed structure variation in these cells from a typical rounded epithelial-like cell to a spindle-shaped with compromised adhesion to the culture surface.

Intermediate cells frequently express staining intensity for kallikreins 8, 13 than mucous cells (7,31,32), a finding that was confirmed by our study. Also, mucous cells had higher staining intensity of kallikrein 10 compared to squamous cells and intermediate cells that is compatible with study of Darling et al.(35). Varying cells of mucoepidermoid carcinoma will not be significant change in the immunohistochemical staining (43). Moreover, Ole-MoiYoi et al. (44) showed that kallikrein 13 was expressed in the epithelium ducts but not in the acinar cells of pancreas and Luo et al.(45) showed that human kallikrein 10 was expression in epithelial ovarian carcinoma.

## Conclusion

Expression of kallikreins 4, 8, 11 and 13 in benign tumors is more than that in normal tissues. In addition, expression of kallikreins 4, 8, 11 and 13 in malignant tumors was more than that in normal control tissues and benign tumors. Kallikreins 4, 11 may be promising new biomarkers in salivary gland tumors. 
